# Complementarity between Early Embryogenesis and Uterine Receptivity: Toward Integrative Approach to Female Infertility Management. Editorial to the Special Issue “Molecular Mechanisms of Human Oogenesis and Early Embryogenesis”

**DOI:** 10.3390/ijms24021557

**Published:** 2023-01-13

**Authors:** Jan Tesarik

**Affiliations:** MARGen Clinic, Molecular Assisted Reproduction and Genetics, Camino de Ronda 2, 18006 Granada, Spain; jtesarik@clinicamargen.com

Two highly relevant papers were recently been published in the Special Issue of *Molecular Sciences* entitled “Molecular Mechanisms of Human Oogenesis and Early Embryogenesis”. Together, these two papers illustrate the interplay between early embryonenesis and uterine receptivity, the two conditions required for the establishment of a pregnancy, each one of them potentiating the other.

The first of these papers reviews the current state of knowledge of the cellular and molecular nature of the fragmentation of human embryos [1]. The other analyzes the multiple effects of the early embryonic product, human chorionic gonadotropin (hCG), on different events related to embryo implantation, including luteal phase support, angiogenetic activity, immunological actions, thyroid function, and the prevention of miscarriage [2].

The former paper [1] shows that embryo fragmentation can be related to various mechanisms, such as apoptotic cell death, the exclusion of altered DNA, and cytoskeletal disorders. These mechanisms can lead to the extrusion of entire blastomeres, apoptotic bodies and micronuclei. It was proposed that, in general, this kind of reaction represents a defense mechanism through which partially damaged embryos try to re-establish their normal developmental pattern by removing aneuploid blastomeres [3].

As early as in 1987, a combined ultrastructural and autoradiographic analysis of human embryos, containing both normal and multinucleated blastomeres and incubated with ^3^H-thymidine or ^3^H-uridine, was performed to evaluate, respectively, the current status of DNA synthesis (replication) and RNA synthesis (transcription) [4]. This study demonstrated the presence of labeled DNA in all nuclei of both normal blastomeres and multinucleated blastomeres. However, nuclei of the multinucleated blastomeres failed to undergo the major activation of RNA transcription, simultaneously occurring in normal blastomeres of the same embryos, and DNA-containing fragments were expelled from the embryos into the perivitelline space [4]. Altogether, these observations suggest that the abnormal (presumably aneuploidic) blastomeres are expelled from the embryo to create free space for the proliferation of normal blastomeres [3].

One important conclusion which can be drawn on the basis of these observations is that the appearance of fragments in the perivitelline space of the human embryo, developing in vitro, should not be considered as a sign of a lack of embryo viability. In fact, it simply means that the embryo has detected an abnormality and is trying to repair it by removing abnormal cells and promoting the proliferation of normal cells. Consequently, actively dividing embryos, though containing fragments, are likely to be more viable than morphologically perfect but developmentally delayed embryos, a condition suggesting that these embryos are unable to repair their abnormalities. One study [5] has suggested that the laser-assisted removal of fragments from the perivitelline space might improve further embryonic development, but further confirmation is needed to determine the truth of this assertion.

The other paper, featured in the same Special Issue [2], highlights the importance of hCG, the early product secreted by the implanting embryo, for the successful initiation of pregnancy. This review article presents a synthesis of all current knowledge on the actions of this multi-effect hormone: the support of the corpus luteum function, angiogenetic activity, immunological actions, and effects on miscarriages and thyroid functions [2]. The paper also analyzes the effects of the four major isoforms of hCG: classical hCG, hyperglycosylated hCG, free β subunit, and sulphated hCG, with respect to the functions outlined above.

Classical hCG is the first molecule synthesized by the human embryo, with the beginning of its RNA transcription as early as the eight-cell stage of embryo development, and the blastocyst produces the protein before implantation [6]. This molecule is essential for the maintenance of the correct function of the corpus luteum and is equally indispensable for implantation and early post-implantation development [7]. The other isoforms are produced later during pregnancy and exert specific effects to maintain pregnancy and prevent spontaneous abortions [2].

Together, the two papers nicely demonstrate the complementarity between embryo health and uterine receptivity. The Special Issue is still open to new contributions. We encourage potential authors to share their experience on this topic.

## References

[1] Cecchele A., Cermisoni G.C., Giacomini E., Pinna M., Vigano P. (2022). Cellular and molecular nature of fragmentation of human embryos. Int. J. Mol. Sci..

[2] d’Hauterive S.P., Close R., Gridelet V., Mawet M., Nisolle M., Geenen V. (2022). Human chorionic gonadotropin and early embryogenesis: Review. Int J. Mol. Sci..

[3] Tesarik J. (2018). Is blastomere multinucleation a safeguard against embryo aneuploidy? Back to the future. Reprod. Biomed. Online.

[4] Tesarík J., Kopecny V., Plachot M., Mandelbaum J. (1987). Ultrastructural and autoradiographic observations on multinucleated blastomeres of human cleaving embryos obtained by in-vitro fertilization. Hum. Reprod..

[5] Rienzi L., Nagy Z.P., Ubaldi F., Iacobelli M., Anniballo R., Tesarik J., Greco E. (2002). Laser-assisted removal of necrotic blastomeres from cryopreserved embryos that were partially damaged. Fertil. Steril..

[6] Bonduelle M.L., Dodd R., Liebaers I., Van Steirteghem A., Williamson R., Akhurst R. (1988). Chorionic gonadotrophin-beta mRNA, a trophoblast marker, is expressed in human 8-cell embryos derived from tripronucleate zygotes. Hum. Reprod..

[7] Tesarik J., Conde-López C., Galán-Lázaro M., Mendoza-Tesarik R. (2020). Luteal phase in assisted reproductive technology. Front. Reprod. Health.

